# Relationship between right-to-left shunt and white matter lesions in patients with migraine: a single-center study

**DOI:** 10.3389/fneur.2024.1419533

**Published:** 2024-08-22

**Authors:** Zhihong Liu, Mingzhu Jiang, Jing He, Yuchan Lin, Lou He, Yan Li, Qi Pan, Shan Wu

**Affiliations:** ^1^Department of Neurology, The Affiliated Hospital of Guizhou Medical University, Guiyang, China; ^2^Department of Neurology, Xiuwen County People's Hospital, Guiyang, China

**Keywords:** migraine, right-to-left shunt, contrast transcranial doppler, cranial magnetic resonance imaging, white matter lesions

## Abstract

**Background:**

Migraine patients have an increased long-term risk of cardio and cerebrovascular events. However, whether these patients are more susceptible to white matter lesions (WMLs) remains debated. To explore this question, our study assessed the proportion of RLS in migraine patients and explored the association between right-to-left shunt (RLS) and WMLs.

**Methods:**

In this study, we included 998 migraine patients. Contrast transcranial doppler (c-TCD) was used to diagnose RLS and assess the extent of the shunt in RLS patients. Of the 998 patients, 505 underwent cranial magnetic resonance imaging (MRI) assessments. WMLs were classified into periventricular white matter lesions (pvWMLs) and deep white matter lesions (dWMLs).

**Results:**

Among the 998 migraine patients, 946 had migraine without aura (MO; mean age 36.68 ± 10.46 years; 80.5% female), and 52 had migraine with aura (MA; mean age 29.85 ± 8.59 years; 71.2% female). Compared with MO patients, MA patients had an earlier onset age (23.1 ± 7.97 vs. 28.44 ± 10.38 years, *p* < 0. 001) and a shorter disease duration (6.76 vs. 8.34 years, *p* = 0.024). The overall proportion of RLS patients was 41.9%, with a greater proportion of RLS patients in the MA group than in the MO group (55.8% vs. 41. 1%, *p* = 0.037). The percentage of RLS-positive patients with no/small shunt was greater in the MO group than in the MA group (81.5% vs. 65.4%, *p* = 0.004), whereas the percentage of RLS-positive patients with moderate/large shunt was greater in the MA group (34.6% vs. 18.5%, *p* = 0.024). The proportion of RLS patients was lower in the WML-positive group (*n* = 173) than in the WML-negative group (*n* = 332), but the difference was not significant (40.5% vs. 45.8%, *p* = 0.253).

**Conclusion:**

This study revealed that 41.9% of migraine patients had RLS, and the proportion of RLS patients was 41. 1% in the MO group and 55.8% in the MA group. The rate of RLS positivity in migraine patients may not be related to the incidence of WMLs.

## Introduction

Migraine is a common primary headache characterized by recurrent unilateral pulsatile, moderate to severe headaches accompanied by neurovegetative symptoms and hypersensitivity to light, sound, and movement. According to the presence or absence of aura symptoms, migraine can be divided into two major types: migraine without aura (MO) and migraine with aura (MA) (1). It is estimated that more than 1 billion people worldwide suffer from migraine, and migraine is considered to be the most common cause of disability in people under 50 years of age (2). Despite the high incidence of migraine, its pathophysiological mechanisms remain unresolved. Patent foramen ovale (PFO), the residual opening of the fetal foramen ovale, is the leading cause of RLS, affecting approximately 20–25% of adults (3–5). Usually, patients with PFO do not show hemodynamic changes during quiet rest, but hemodynamic changes occur after some vigorous activities, such as coughing, sneezing, and Valsalva movements, due to right-to-left PFO shunting. Previous studies have shown that PFO is closely related to migraine, and the prevalence of PFO in migraine patients is approximately 46.3–88%, especially in patients with MA (6). Additionally, numerous previous studies have demonstrated a high prevalence of white matter lesions (WMLs) in individuals with migraine. Compared with nonmigraine controls, adult migraineurs are at a two-to four-fold increased risk of developing WMLs (7).

Some researchers have suggested that RLS may be involved in the development of WMLs in individuals with migraine, especially those who experience migraine with aura (8). However, in a recent study exploring the relationship between cerebral hemodynamics, RLS, and WMLs in patients with MA, young stroke patients, and control groups, the authors found that the presence of RLS was likely not involved in the genesis of WMLs in MA patients (9). In addition, a Mendelian randomization study to investigate the bidirectional causal relationship between migraine and WMLs at the genetic level found no evidence of a causal relationship between migraine and white matter lesions (10). As a result, research on the association between migraine, RLS, and white matter lesions has produced conflicting results.

To explore this question, this study conducted the following assessments: (1) describing the proportion of RLS in migraine patients; (2) exploring the association between RLS and WMLs in migraine patients.

## Methods

### Patients

In this study, a total of 11,555 patients who underwent c-TCD in the Department of Neurology at the Affiliated Hospital of Guizhou Medical University between January 2020 and August 2023 were retrospectively included. We examined the patients’ electronic medical records and included 998 migraine patients according to the diagnostic criteria in the International Classification of Headache Disorders, 3rd edition (1). The selected individuals were categorized into MA and MO groups. In the second part of the study, 505 migraine patients were included, while 493 patients were excluded due to (1) previous symptomatic stroke, (2) other types of primary headache, (3) demyelinating disease or hereditary white matter disease, (4) tumors, (5) intracranial vascular stenosis or occlusion, (6) intracranial organic disease, or (7) failure to complete cranial MRI. Age, sex, past history, duration of migraine, age at first onset, presence of aura, and family history of migraine were collected for all eligible patients. This study was approved by the Ethics Committee of the Affiliated Hospital of Guizhou Medical University (approval number: 2024 ethics review no. 85; registered on February 4, 2024).

### c-TCD examination

**Figure 1:**
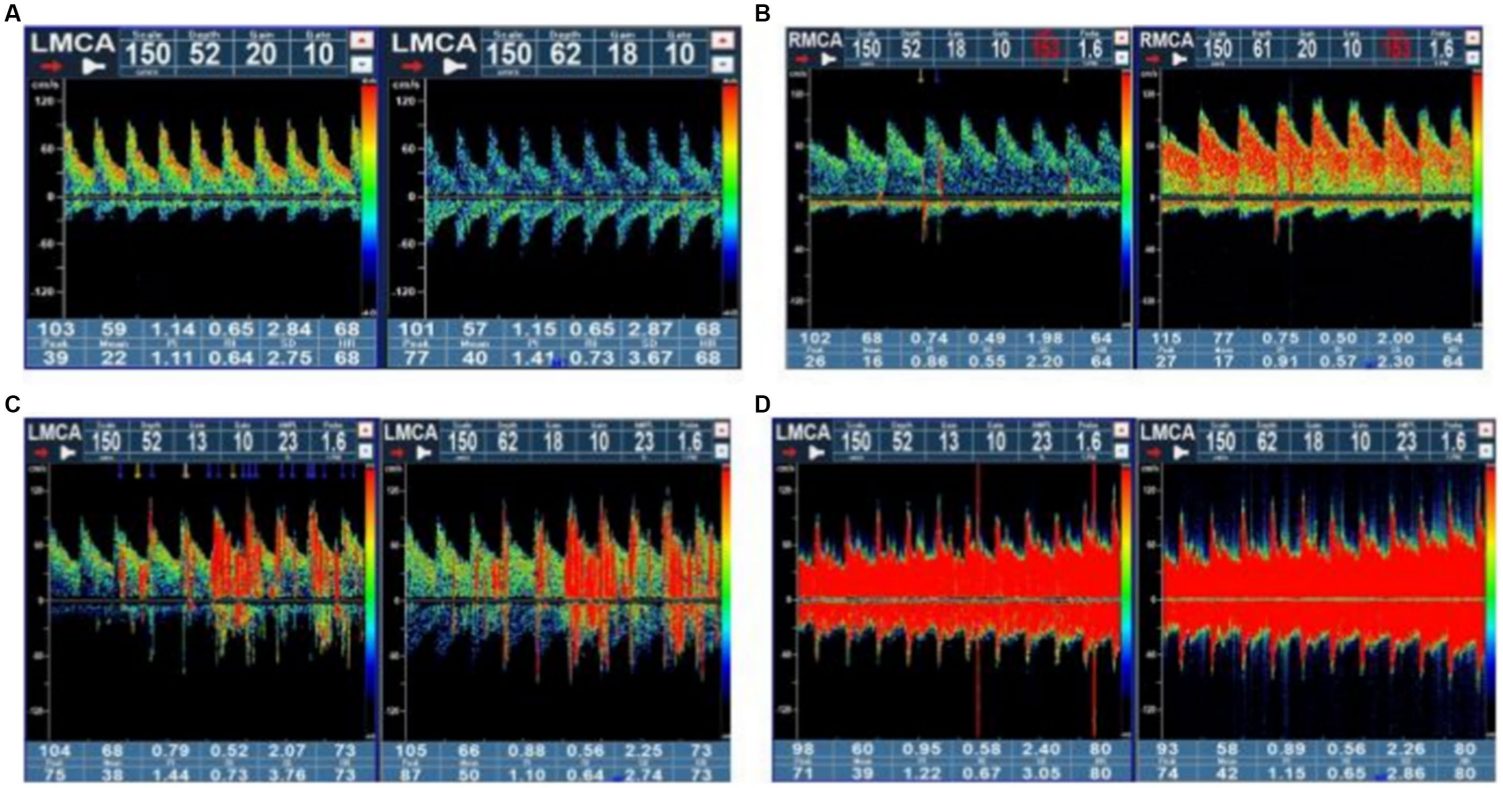
Four-level RLS categorization based on the microbubble count. Grade 0 = negative; Grade I = 1–10 MBs; Grade II = more than 10 MBs but no rain curtain; Grade III = formation of a rain curtain. RLS, right-to-left shunt; MBs, microbubbles.

c-TCD examinations were performed with a handheld 2-MHz probe connected to the TCD detector (EMS-9 PB, Delica, China). We chose a single-channel dual-depth mode to observe one side of the middle cerebral artery (MCA). If ultrasound was unable to penetrate the temporal window for monitoring the MCA, an alternative option was to monitor the vertebrobasilar artery through the occipital window. Before the test, patients were asked to practice a standardized Valsalva maneuver. Patients were in a supine position on the examination bed. Two 10 mL syringes were prepared: one filled with 8 mL of normal saline and 1 mL of air, and the other filled with 1 mL autologous venous blood. A three-way tube was connected to the patient’s venous access. Then, the two syringes were alternately injected back and forth 20–30 times to create the contrast agent. The patients were immediately given a “bullet-like” injection while in a resting state, and the presence of microbubble signals was recorded within 25 s. The patients rested flat for 2 min, during which time the contrast agent was prepared again. During the second injection, the patients were instructed to perform the Valsalva maneuver. The Valsalva maneuver should be initiated at 5 s after injection of the contrast agent and maintained for 10 s. The standard for an effective Valsalva maneuver was a reduction of at least 25% of the mean MCA velocity, and the presence of microbubble signals was recorded within 25 s. The above tests (including at rest and under provocative conditions) were conducted 2 to 3 times. Microbubbles (MBs) in the resting state or after the Valsalva maneuver indicated positivity for RLS; negativity for RLS was indicated if MBs were not detected. According to the grading criteria established by the Chinese Society of Cardiology (11), a four-level RLS categorization, based on the MB count, was applied as follows: Grade 0 = negative; Grade I = 1–10 MBs; Grade II = more than 10 MBs but no rain curtain; Grade III = formation of a rain curtain (Figure 1). In this study, Grade 0 was defined as no diversion, Grade I as a small diversion, Grade II as a moderate diversion, and Grade III as a large diversion. (The source of the following image: Provided by the Transcranial Doppler Examination Room of the Neurology Department, Affiliated Hospital of Guizhou Medical University.)

### MRI

**Figure 2:**
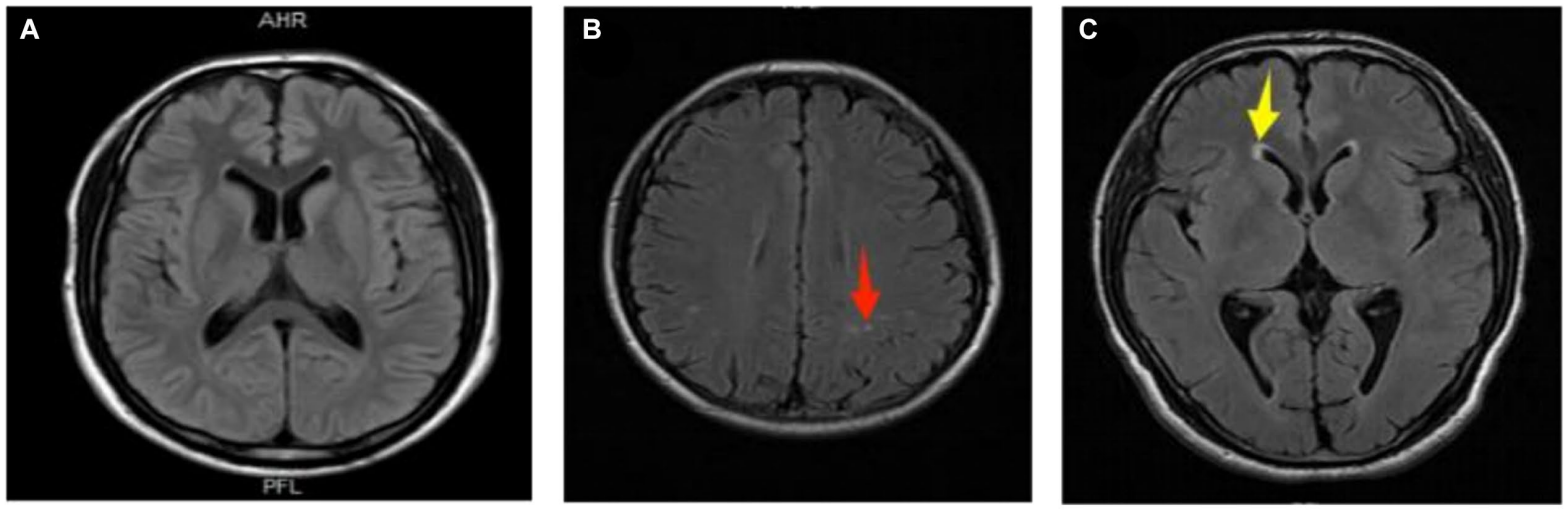
Representative images of white matter lesions. **(A)** Normal; **(B)** deep white matter lesion (dWMLs), multiple clear boundary signals observed in the bilateral radiative coronal region (red arrow); **(C)** paraventricular white matter lesion (pvWMLs), a hat-shaped high signal observed in the anterior horn of the right lateral ventricle (yellow arrow).

In this study, cranial MRI was performed by Siemens 3.0 T magnetic resonance imaging equipment. T1-weighted sequence (T1WI), T2-weighted sequence (T2WI) and fluid attenuated inversion recovery (FLAIR) images were collected with a layer thickness of 5 mm and a layer spacing of 6 mm. Two experienced neurologists assessed WMLs based on consensus, without access to the clinical information. An image processing workstation was used to assess WMLs on T1/T2/FLAIR images in the enrolled group. A WML is defined as isointensity or slight hypointensity on T1-weighted image sequences (T1WI), slight hyperintensity on T2-weighted images (T2WI), and hyperintensity on fluid-attenuated inversion recovery sequence images (T2-FLAIR), with clear boundaries from the surrounding tissue and no occupancy effect (12). The white matter lesions were categorized into paraventricular white matter lesions (pvWMLs) and deep white matter lesions (dWMLs) based on their location. The former was located near the anterior horn, posterior horn, or body of the lateral ventricle. The latter was located in the white matter region of the deep white matter and was not connected to the lateral ventricle lesion. The white matter lesions on MRI were punctate white matter lesions, which were not applicable to the Fazekas scale. We chose typical images for Figure 2. (The source of the following image: Provided by the Department of Radiology, Affiliated Hospital of Guizhou Medical University.)

### Statistical analysis

This study used SPSS (IBM, version 26.0, IBM Corporation, Armonk, NY, United States) for statistical analysis, and *p* < 0.05 was defined as having statistical significance. The Chi-square test and Independent-sample t test were used to compare demographics and baseline characteristics among different subtypes of migraine patients, as well as the cranial MRI results. Using logistic regression analysis, after adjusting for confounding factors such as age, gender, disease duration, presence of aura, and family history, the correlation between RLS grade and white matter lesion classification was analyzed.

## Results

### Clinical characteristics of patients with different migraine subtypes

**Figure 3:**
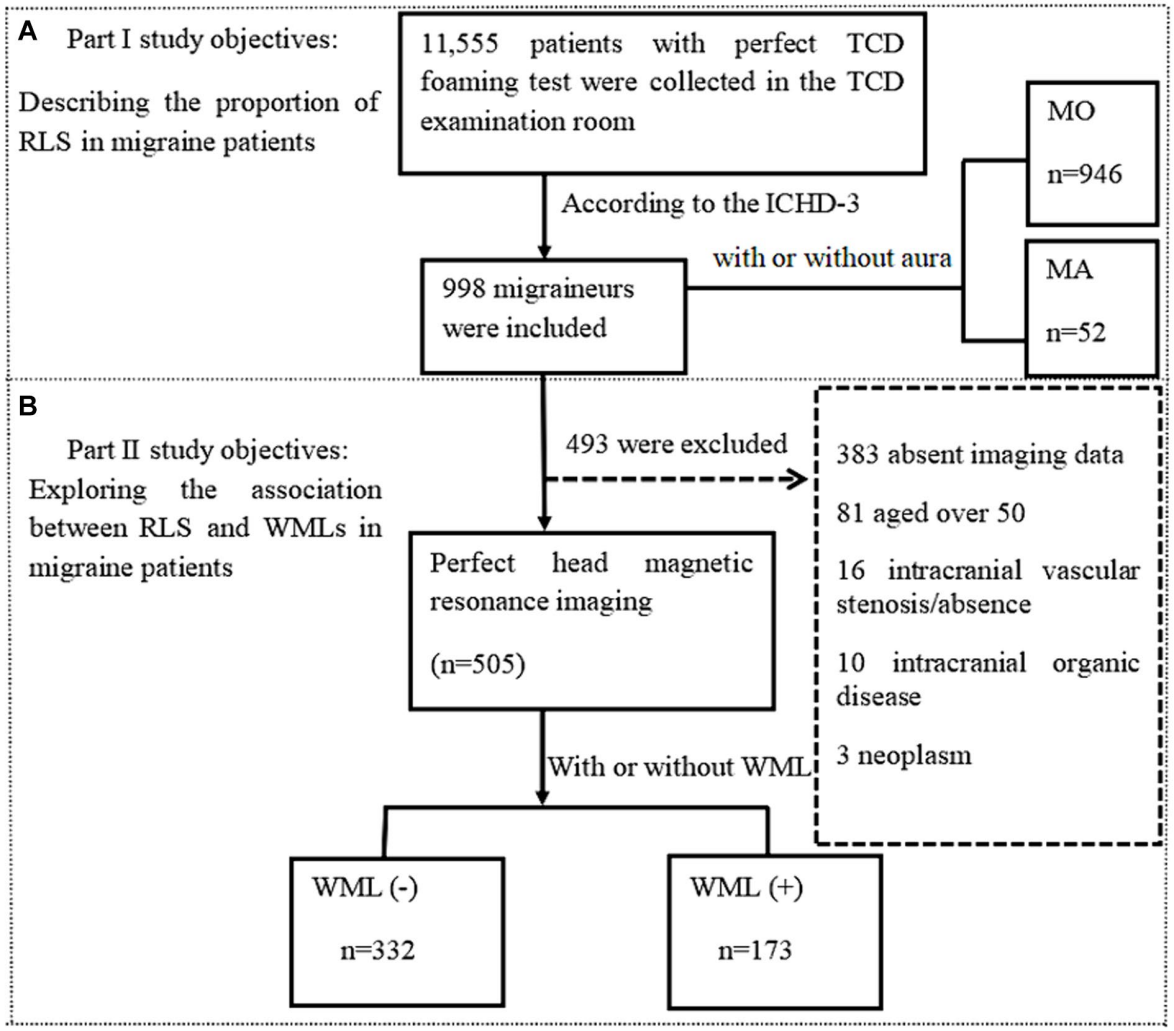
Flow chart of patient enrollment.

A total of 998 patients with migraine (799 females, 199 males; mean age: 30.89 ± 10.98 years) were included according to the International Classification of Headache Disorders-3 beta (ICHD-3) diagnostic criteria (1) (Figure 3). Among them, 52 patients (5.2%) were diagnosed with MA and 946 patients (94.8%) were diagnosed with MO. MA patients were found to be younger (29.85 vs. 36.68 years, *p* < 0 0.001), had an earlier age at first onset (23.1 vs. 28.44 years, *p* < 0.001), and had a shorter disease duration (6.76 vs. 8.34 years, *p* = 0.024) compared to MO patients. Among all migraineurs, RLS was present in 41.9% (418/998). The proportion of RLS patients was lower in the MO group than in the MA group, and the difference was significant (41.1% vs. 55.8%, *p* = 0.037). The positive rate of RLS was higher in the MO group than in the MA group for no/small shunt (81.5% vs. 65.4%, *p* = 0.004), but lower in the MO group than in the MA group for moderate/large shunt (18.5% vs. 34.6%, *p* = 0.024) (Table 1).

**Table 1:** Comparison of the clinical characteristics between MA and MO patients.

Clinical data	MO (*n* = 946)	MA (*n* = 52)	t/x^2^	*p*-value
Age (years)	36.68 (10.46)	29.85 (8.59)	5.515	**<0.001**
Female, *n* (%)	762 (80.5)	37 (71.2)	2.726	0.099
Hypertension, *n* (%)	68 (7.2)	1 (1.9)	2.123	0.145
Diabetes, *n* (%)	10 (1. 1)	0 (0)	0.555	0.456
Dyslipidemia, *n* (%)	115 (12.2)	5 (9.6)	0.301	0.583
Arrhythmia, *n* (%)	9 (1.0)	0 (0)	0.499	0.480
Smoking, *n* (%)	17 (1.7)	0 (0)	0.951	0.330
Drinking, *n* (%)	8 (0.8)	1 (1.9)	0.640	0.424
Family history of migraine, *n* (%)	197 (20.8)	13 (25.0)	0.517	0.472
Duration of migraine, *n* (years)	8.34 (7.66)	6.76 (4.58)	2.313	**0.024**
Age at first onset	28.44 (10 0.38)	23. 1 (7.97)	4.574	**<0.001**
RLS positive, *n* (%)	389 (41. 1)	29 (55.8)	4.345	**0.037**
**RLS grade**				
0-I (no/small shunt)	771 (81.5)	34 (65.4)	8.207	**0.004**
II-III (moderate/large shunt)	175 (18.5)	18 (34.6)	8.201	**0.024**

Grade 0 was defined as no diversion, Grade I as a small diversion, Grade II as a moderate diversion, and Grade III as a large diversion. O, migraine without aura; MA, migraine with aura; RLS, right-to-left shunt; WMLs, white matter lesions. The bolded values represent *p*-values less than 0.05.

### Proportion and characteristics of RLS in patients with different migraine subtypes

**Figure 4:**
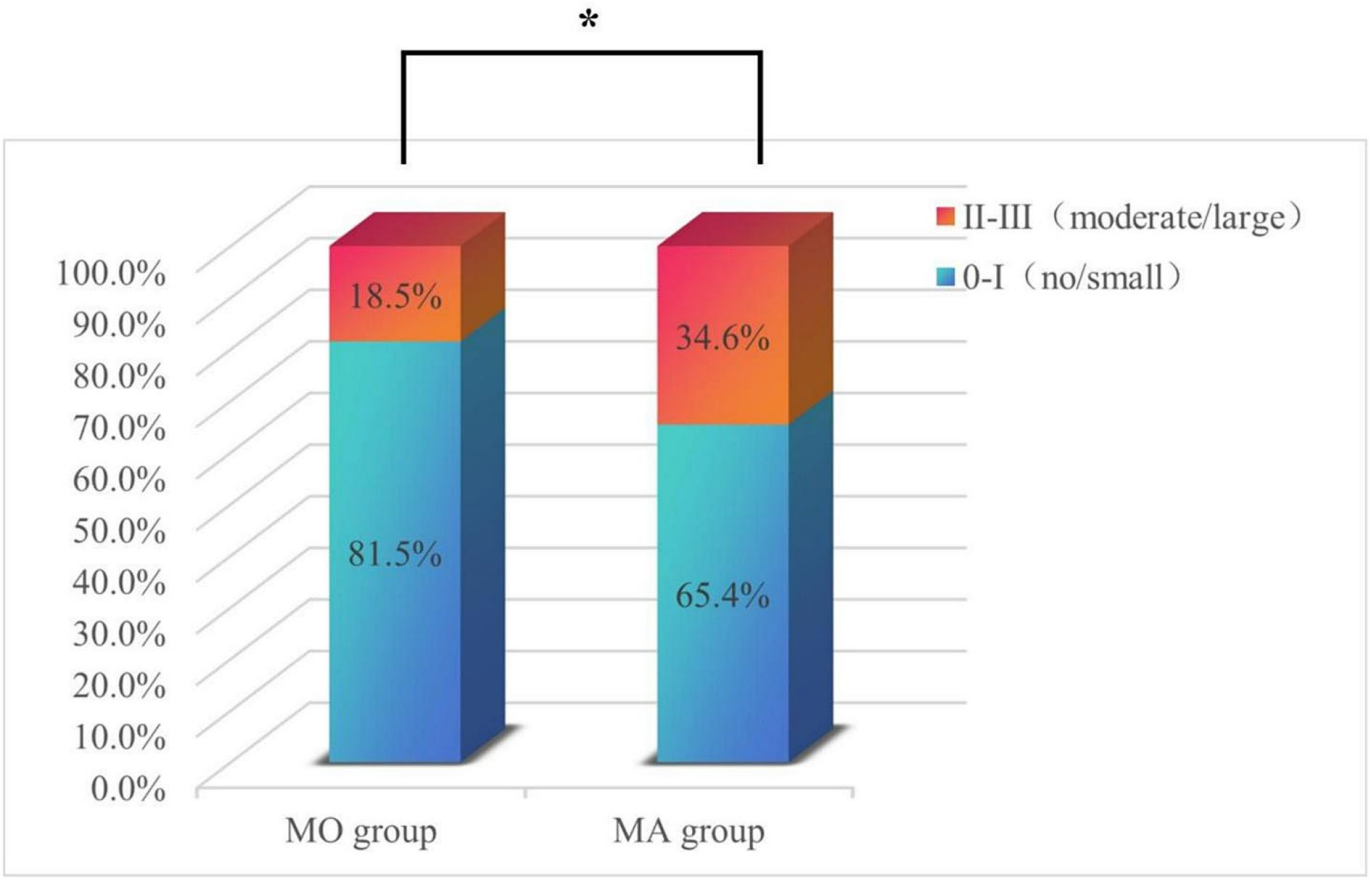
The proportions of patients with no/small RLS and moderate/large RLS in the MO and MA subgroups. The percentage of patients with no/small shunt in the MO group was significantly greater than that in the MA group; the percentage of patients with a moderate/large shunt in the MA group was significantly greater than that in the MO group. *p < 0.05.

Figure 4 shows that the MA group exhibited a higher overall proportion of RLS patients compared to the MO group (55.8% vs. 41. 1%, *p* = 0.037). Additionally, patients in the MA group had a higher proportion of moderate/large shunt than those in the MO group (34.6% vs. 18.5%, *p* = 0.024). Conversely, the proportion of no/small shunt was higher in the MO group than in the MA group (81.5% vs. 65.4%, *p* = 0.004).

### Comparison of characteristics between WML-negative and WML-positive groups

To explore the relationship between RLS and positive WMLs in migraine patients, we screened 505 out of 998 migraineurs based on the aforementioned inclusion and exclusion criteria (Figure 3). The mean age of the patients was 34.04 ± 8.45 years, and the mean disease duration was 7.44 ± 6.82 years, with females accounting for 80%. Among them, 332 (65.7%) were WML-negative and 173 (34.3%) were WML-positive. The WML-positive group was significantly older than the WML-negative group (36.66 ± 8.56 vs. 32.67 ± 8.08 years, *p* < 0.001). Patients in the WML-positive group had a longer disease duration compared to the WML-negative group (8.34 ± 7.78 vs. 6.97 ± 6.22 years, *p* = 0.046). Additionally, patients in the WML-negative group had a younger age at first onset than those in the WML-positive group (25.72 ± 8.42 vs. 28.43 ± 9.81 years, *p =* 0.002). There were no significant differences in gender distribution, vascular risk factors, family history of migraine, or migraine with aura between the two groups.

The prevalence of RLS was lower in the WML-positive group than in the WML-negative group (40.5% vs. 45.8%, *p* = 0.253). The incidence of WML did not increase with increasing RLS grade (*p* = 0.596) (Table 2).

**Table 2:** Comparison of characteristics between the WML-negative and WML-positive groups.

Characteristics	WML (−)	WML (+)	t/x^2^	*p*-value
Age (years)	32.67 (8.08)	36.66 (8.56)	−5.162	**<0.001**
Female, *n* (%)	266 (80. 1)	138 (79.8)	0.009	0.925
Diabetes, *n* (%)	4 (1.2)	1 (0.6)	0.456	0.500
Hypertension, *n* (%)	21 (6.3)	16 (9.2)	1.431	0.232
Dyslipidemia, *n* (%)	45 (13.6)	26 (15.0)	0.205	0.651
Arrhythmia, *n* (%)	3 (0.9)	1 (0.6)	0.153	0.695
Smoking, *n* (%)	5 (1.5)	3 (1.7)	0.038	0.846
Drinking, *n* (%)	4 (1.2)	1 (0.6)	0.456	0.500
Family history of migraine, *n* (%)	81 (24.4)	36 (20.8)	0.823	0.364
Duration of migraine, *n* (years)	6.97 (6.22)	8.34 (7.78)	−2.000	**0.046**
Age at first onset	25.72 (8.42)	28.43 (9.81)	−3.088	**0.002**
Migraine with aura	23 (6.9)	11 (6.4)	0.059	0.809
Migraine without aura	309 (93. 1)	162 (93.6)		
RLS positive, *n* (%)	152 (45.8)	70 (40.5)	1.307	0.253
**RLS grade**				
0	180 (54.2)	103 (59.5)	1.889	0.596
I	86 (25.9)	38 (22)		
II	42 (12.7)	18 (10.4)		
III	24 (7.2)	14 (8. 1)		

RLS, right-to-left shunt; WMLs, white matter lesions. The bolded values represent *p*-values less than 0.05.

### Correlation between RLS grade and white matter lesion classification

To further investigate the correlation between RLS and WML positivity in migraine patients, we performed a logistic regression model analysis (Table 3). The results showed that when controlling for the correlation analysis of multiple factors such as age, gender, disease duration, aura, and family history in binary logistic regression analysis, migraine patients were classified according to RLS grading (0-III). Compared with RLS Grade 0 subjects, the likelihood of developing white matter lesions in RLS Grades I, II, and III did not significantly improve or decrease. Furthermore, we further divided WMLs into pvWML, dWML, and pvWML+dWML groups. The results showed that subjects with RLS Grade I were less likely to develop deep white matter lesions compared to those with RLS Grade 0 (8.9% vs. 8.1%, *p* = 0.042; OR = 0.597; 95% CI: 0.363–0.981).

**Table 3:** Correlation between RLS grade and white matter lesion classification.

	WML		pvWML		dWML		pvWML + dWML	
	*n* (%)	OR (95% CI)	*n* (%)	OR (95% CI)	*n* (%)	OR (95% CI)	*n* (%)	OR (95% CI)
Grade 0 *n* = 283	103 (36.4)	—	96 (33.9)	—	23 (8. 1)	—	16 (5.7)	—
Grade I	38 (30.6)	0.760 (0.416–1.212)	30 (24.2)	1.097 (0.512–2.553)	11 (8.9)	0.597 (0.363–0.981)	3 (2.42)	0.382 (0.107–1.369)
*n* = 124		*p* = 0.249		*p* = 0.811		***p* = 0.042**		*p* = 0.140
Grade II	18 (30.0)	0.695 (0.372–1.295)	15 (25.0)	0.765 (0.251–2.330)	4 (6.7)	0.586 (0.304–1.717)	11 (16.7)	0.239 (0.030–1.889)
*n* = 60		*p* = 0.252		*p* = 0.638		*p* = 0.111		*p* = 0.175
Grade III	14 (36.8)	0.925 (0.446–1.918)	13 (34.2)	1.578 (0.551–4.519)	5 (13.2)	0.898 (0.426–1.896)	4 (10.5)	1.574 (0.464–5.338)
*n* = 38		*p* = 0.834		*p* = 0.396		*p* = 0.779		*p* = 0.467

The *p* value indicates the possibility of white matter lesions in subjects with RLS levels I, II, and III compared to those with RLS level 0. WML: brain white matter lesion; pv WML: paraventricular white matter lesion; d WML: deep brain white matter lesion.

## Discussion

In our study, we investigated the prevalence of RLS in migraine patients and assessed whether RLS was associated with a higher prevalence of white matter hyperintensities. Consistent with previous studies, we found that the RLS rate was 41.9% in migraine patients and 55.8% in migraine with aura patients. In a Japanese study of 112 migraine patients, the overall prevalence of RLS in migraine patients was 54.5%, with a higher proportion of 62.9% in the MA group (13). In an Italian study, the prevalence of RLS was 61.9% in the migraine with aura group and 16.2% in the migraine without aura group among 334 participants (14). In comparison to the previous research, our study includes a larger sample of migraine patients. Our findings confirm a high prevalence of RLS in migraine patients, particularly those with aura.

Furthermore, we observed the relationship between RLS size and migraine. A previous study demonstrated that Valsalva-induced RLS, particularly large RLS, was more prevalent in migraine with aura patients (60%; OR = 2.3; 95% CI: 1.2–4.3; *p* = 0.01) than in migraine without aura patients (40%; OR = 2.3; 95% CI: 1.2–4.3; *p* = 0.01) (15). Our study also found that migraineurs with aura were more likely to have moderate/large RLS (34.6% vs. 18.5%, *p* = 0.004), which was consistent with the results of a multicenter case—control study in China (13). These findings imply a potential involvement of RLS in the pathogenesis of migraine. Nevertheless, the underlying mechanism linking RLS and migraine remains unclear. First, some scholars have proposed that due to the presence of patent foramen ovale, vasoactive substances can bypass the pulmonary circulation and directly cross the blood–brain barrier to induce headaches (16). This hypothesis has been supported by certain studies. One clinical study found that peripheral blood serotonin expression levels in patients undergoing RLS occlusion were significantly reduced by 27.27% during follow-up compared with patients treated with medication alone (*p* = 0.0034) (17). Second, microemboli from the venous system enter the arterial system directly through the RLS when the right atrial pressure temporarily increases. This “paradoxical embolism” causes minor cerebral infarction, triggering hypoperfusion or cortical spreading depression (CSD) and leading to migraine attacks. This is the most likely pathological and physiological basis for RLS induced migraine attacks (18). An animal study showed that an intracranial injection of air to form microemboli in migraine model mice can induce cortical diffusion inhibition without causing ischemic stroke (19). Third, due to the presence of RLS, nonoxygenated venous blood directly entering the systemic circulation can cause transient hypoxemia, which directly triggers the occurrence of migraine (20). Some authors demonstrated the significant involvement of the NOTCH3 gene in the closure process of the foramen ovale (21).

Previous studies have shown that there is a close relationship between migraine and white matter hyperintensities, but the mechanism remains unclear. In a multicenter study of 334 Chinese migraine patients, the prevalence of WMLs was found to be 52.7% (176/334), and this prevalence was not influenced by the patients’ course (21). In our study, we observed a WML incidence of 34.3% (173/505) among migraineurs. Patients in the WML-positive group were found to have a longer duration of migraine than patients in the WML-negative group, and the difference was significant. Compared to this multicenter study of 334 migraine patients in China, the prevalence of WMLs in migraine patients at our center was lower (22). It may be caused by the different age composition ratio of our participants. The mean age of patients in our study was 34.04 ± 8.45 years, while in a study by Jiang et al., the mean age of patients was 44.03 ± 10.12 years (22), and the incidence of WMLs was less than 30% of people under 50 years of age and without cerebrovascular risk factors (23). This may also be related to the low proportion of migraine patients with aura in our study, as previous studies have shown that MA is a risk factor for WMLs. In a follow-up study, 41 MA patients were followed for an average of 33.2 months. The findings indicated a progressive increase in the number of white matter lesions over time, and this progression may be linked to longer duration of aura (*r* = 0.60, *p* < 0.0001), suggesting potential brain tissue damage when local reduction of cerebral blood flow reaches the threshold for tissue damage during migraine attacks with aura (24).

The relationship between WMLs and RLS in migraine patients is unknown thus far. In 2010, Park et al. included 242 migraine patients in their study, and the results indicated that the presence of RLS was an independent predictive factor for the prevalence of small rather than moderate or large WMLs (25). It was speculated that paradoxical emboli caused by RLS may mainly lead to small WMLs in young migraine patients.

In 2017, Iwasaki’s study showed that 59 out of 107 migraine patients had aura, demonstrating a possible association between RLS and WMLs in migraine patients (26). In 2022, Huo et al. included 201 migraine patients, and the results showed that near-cortical WMLs in migraine patients were related to RLS, especially in the blood supply area of the anterior cerebral artery. It is speculated that white matter lesions near the cortex may be caused by paradoxical embolism mechanisms, as microemboli are more likely to enter the distribution area of the anterior cerebral artery, which runs straighter than other blood vessels, through RLS when the right atrial pressure increases (27). In 2023, a study with 334 migraine patients showed that the positive rate of RLS was related to the incidence of WMLs, but different RLS levels were not related to the severity of WMLs (28).

Some studies have reported opposite results. In 2007, Del Sette et al. (29) recruited 87 migraine patients with aura, but the results showed that the number and volume of WMLs in migraine patients with aura were not related to the presence of RLS. In addition, Koppen et al. (14) included 166 migraine patients, and the results showed that RLS was not related to the high incidence rate of WMLs in migraine patients. Our research results are consistent with theirs. In our study, we further divided WML positive patients into dWML and pvWML groups. Logistic regression analysis was adjusted for age, gender, disease duration, presence of aura, and family history. We found that a small shunt was associated with dWMLs and was a protective factor for dWMLs (OR = 0.597, 95% CI 0.363–0.981, *p* = 0.042). This discrepancy may be attributed to the small sample size of dWML cases (*n* = 20), necessitating further support from larger samples in subsequent studies. Overall, the different outcomes of these studies may be related to different study designs, participant characteristics, diagnostic determination methods (diagnosis through medical interviews and self-report), statistical methods for follow-up, and neuroimaging methods (especially lack of standardization, such as high and low magnetic fields, scanner types, and selection of sequence or slice thickness). It should also be noted that the sensitivity of using 1.5-T or 3.0-T MRI to detect WMLs may be higher than that of detecting cortical lesions.

Our study includes the following limitations. First, because this was a single-center study, there may be a selection bias on the data. The sample size of MA subjects in a single center is limited, and the variation in the prevalence of RLS may not significantly impact the overall association between RLS and MA. Second, the retrospective nature of this study leads to a lot of unpredictable data loss. In this study, the fact that the number of migraine patients with aura was lower than expected compared to the total number of patients should be considered as a disadvantage. Third, the lack of a control group should also be considered as a weakness due to the retrospective nature of the study. Therefore, this study could not identify differences in the prevalence and extent of RLS between migraine patients and healthy subjects. Fourth, this study focused only on the presence and location of WMLs without measuring the number and burden of lesions. Fifth, this study did not utilize transesophageal echocardiography (TEE) to determine PFO, only c-TCD. As RLS is divided into both intracardiac RLS (such as PFO) and extracardiac RLS (pulmonary arteriovenous fistula), further research is needed to explore the proportion of PFO and pulmonary arteriovenous obstruction patients. Finally, this study was not a longitudinal study. It is necessary to conduct longitudinal studies to determine whether RLS is a risk factor for WMLs in migraine patients.

## Conclusion

Our study showed a right-to-left shunt in 41.9% of migraineurs, with a prevalence of RLS of 41. 1% in migraineurs without aura and 55.8% in migraineurs with aura. The positive rate of RLS in migraine patients may not be related to the incidence of WMLs.

## Data Availability

The original contributions presented in the study are included in the article/supplementary material, further inquiries can be directed to the corresponding author.
