# Corrigendum: Relationship between right-to-left shunt and white matter lesions in patients with migraine: a single-center study

**DOI:** 10.3389/fneur.2024.1509631

**Published:** 2024-10-30

**Authors:** Zhihong Liu, Mingzhu Jiang, Jing He, Yuchan Lin, Lou He, Yan Li, Qi Pan, Shan Wu

**Affiliations:** ^1^Department of Neurology, The Affiliated Hospital of Guizhou Medical University, Guiyang, China; ^2^Department of Neurology, Xiuwen County People's Hospital, Guiyang, China

**Keywords:** migraine, right-to-left shunt, contrast transcranial doppler, cranial magnetic resonance imaging, white matter lesions

In the published article, there was an error regarding the affiliation(s) for Zhihong Liu. As well as having affiliation Number 2 (Department of Neurology, Xiuwen County People's Hospital, Guiyang, China), she should also have affiliation Number 1 (Department of Neurology, The Affiliated Hospital of Guizhou Medical University, Guiyang, China).

Please change the affiliation Number 1 to “Department of Neurology, The Affiliated Hospital of Guizhou Medical University, Guiyang, China”, and affiliation Number 2 to “Department of Neurology, Xiuwen County People's Hospital, Guiyang, China”.

Please change the superscript numbers from affiliation Number 2 to 1 for the names Mingzhu Jiang, Jing He, Yuchan Lin, Lou He, Yan Li, Qi Pan and Shan Wu.

Final version:

Zhihong Liu^1, 2^, Mingzhu Jiang^1^, Jing He^1^, Yuchan Lin^1^, Lou He^1^, Yan Li^1^, Qi Pan^1*^ and Shan Wu^1*^

^1^Department of Neurology, The Affiliated Hospital of Guizhou Medical University, Guiyang, China

^2^Department of Neurology, Xiuwen County People's Hospital, Guiyang, China

The authors apologize for this error and state that this does not change the scientific conclusions of the article in any way. The original article has been updated.

